# Annotations

**Published:** 1906-02-10

**Authors:** 


### ANNOTATIONS.

### The Plague of Sweets.

An inquest was held at Eccles on Monday respecting the cause of death in the case of two boys of five and six. The children, who died on Sunday very suddenly, had been given a halfpenny on the previous day with which they purchased two chocolate wafers. In the evening they began to vomit, and the medical man who was called in the next day discovered signs of irritant poisoning. Before night they expired, and at the inquest it was decided to hold a post-mortem and to analyse the biscuits. It transpired in evidence that eighteen of the wafers had been sold from the same tin, and that, as far as it was known, no other instance of the consumers having been poisoned was forthcoming. This may either mean that the rest of the consumers were not so susceptible to the irritant, or that the deaths were due to another cause. But, however that may be, the incident serves as a reminder of the ever-increasing mischief wrought in this country by the inordinate consumption of sweets. We are not at all sure that the evil consequences to health are not as great as those from the sale of intoxicating liquors. The amazing thing is, not so much that grown-up people munch chocolate in all parts of the theatre and that every young maidservant on her evening out treats herself to caramels or comfits, but that persons who cannot afford to give their children sufficient bread, find regularly each week a copper for the cheap confections displayed in the windows of multitudinous shops which, for some occult reason, are allowed to be open on Sundays as well as week days. If not poisonous, the sweets are often cheap and nasty, they are the source of all sorts of unsuspected ailments, they are ruinous to a healthy appetite, and in the aggregate a vast sum of money is wasted upon them which might be devoted to the purchase of badly needed food. We are afraid that the plague will not be easily stayed, but it would, perhaps, be mitigated by a material augmentation in the price of sugar, if our new Chancellor of the Exchequer has the courage to face a little passing odium.

### Compulsory Military Training.

Lord Roberts is fitting an appropriate copingstone to his life of adventure and daring, and we doubt whether any of the fine achievements which have made his name honoured throughout the world has instanced as fine a courage as his present campaign against the coma which embraces our national patriotism at this moment. A century of security if it has not sapped our energies has at least rocked them to sleep, and the *vis inertiæ* of forty million sleepers is not easily overcome. Yet this is the task to which the veteran field-marshal has elected to devote those years which most men are content to devote to repose, and the enthusiasm with which he has prosecuted his crusade in the teeth of an apathy which is admittedly harder to bear than is active antagonism bears superfluous witness both to his patriotism and pluck. The object of his crusade, whatever be its value as an element of national safety, is incontestably one which must command the support of all those who, like the members of the medical profession, are keenly interested in the physical and moral education of the race. Those who see much of the poorest classes in great cities can hardly fail to be impressed with the blighting effects which lack of physical culture and lack of discipline are capable of exercising upon children, even of such a fine stock as ours. Any system, therefore, which will make compulsory methods of greater care in the physical and moral (as opposed to the intellectual) education of the masses must command the acceptance of all thinking people. There is a consensus of opinion that conscription as practised on the Continent has done much to raise the physical standard of those races which employ it, and it seems probable that a similar result can be achieved under the scheme of Lord Roberts at a far smaller sacrifice than is involved in conscription. The weight of medical opinion is gradually growing in the councils of the State; let us see that it is cast into the right scale and in good time.

### Glasgow's Way with Drunkards.

The Glasgow municipality has prepared a Bill the object of which is to amend the existing Inebriates Acts. At present, before an inebriate can be sent to a reformatory without his own consent, he must have been convicted in a police court four times within a year. It is obvious that an inebriate may have become hopelessly demoralised long before he comes within the purview of the police. The method proposed in the Glasgow Bill is something akin to the powers possessed in France by the *conseil de famille*. A parent, husband, wife, or adult child may, having obtained the previous consent of two magistrates or justices of the peace, present a petition to the sheriff of the county in which he resides, setting forth that the person referred to is "a habitual drunkard and that he is, by reason of his conduct and habits of life, a fit and proper subject for treatment in an inebriate reformatory." On receipt of this petition the sheriff may, after such inquiry as he deems necessary, grant a warrant to cite such person to appear for trial before him with a jury. The jury then decide from the evidence brought before them whether or not the person cited is a fit subject for treatment, and if they decide in the affirmative, the sheriff may order his detention. This procedure seems elaborate, and one fears that it may be too troublesome, even if not too expensive, for many of those who most need the protection it will give from the evil done by a drunken husband or wife. An elaborate procedure is however, necessary, to prevent the detention of anyone on account of the mere malice of relatives. But no one can doubt that in the majority of cases where parent, husband, wife, or child brings the accusation forward, they are driven to it by the conduct of the relative in question, and certainly the abandonment of the conviction in a police court is a forward step towards the reclamation of the inebriate while there is still a fair hope of improvement.

